# LMI-Based H_∞_ Controller of Vehicle Roll Stability Control Systems with Input and Output Delays

**DOI:** 10.3390/s21237850

**Published:** 2021-11-25

**Authors:** Jonatan Pajares Redondo, Beatriz L. Boada, Vicente Díaz

**Affiliations:** Mechanical Engineering Department, Institute for Automotive Vehicle Safety (ISVA), Universidad Carlos III de Madrid, Avda. de la Universidad 30, Leganés, 28911 Madrid, Spain; bboada@ing.uc3m.es (B.L.B.); vdiaz@ing.uc3m.es (V.D.)

**Keywords:** networked control systems, input and output delay, H_∞_ controller, vehicle dynamics, roll stability control

## Abstract

Many of the current research works are focused on the development of different control systems for commercial vehicles in order to reduce the incidence of risky driving situations, while also improving stability and comfort. Some works are focused on developing low-cost embedded systems with enough accuracy, reliability, and processing time. Previous research works have analyzed the integration of low-cost sensors in vehicles. These works demonstrated the feasibility of using these systems, although they indicate that this type of low-cost kit could present relevant delays and noise that must be compensated to improve the performance of the device. For this purpose, it is necessary design controllers for systems with input and output delays. The novelty of this work is the development of an LMI-Based H${}_{\infty }$ output-feedback controller that takes into account the effect of delays in the network, both on the sensor side and the actuator side, on RSC (Roll Stability Control) systems. The controller is based on an active suspension with input and output delays, where the anti-roll moment is used as a control input and the roll rate as measured data, both with delays. This controller was compared with a controller system with a no-delay consideration that was experiencing similar delays. The comparison was made through simulation tests with a validated vehicle on the TruckSim^®^ software.

## 1. Introduction

Many of the current research works are focused on the development of different control systems for commercial vehicles in order to significantly reduce the incidence of dangerous driving situations and improve vehicle stability and comfort. One of the accidents with the worst consequences in heavy-duty vehicles is a rollover. For that reason, previous works [1,2,3] have used different methods such as differential braking, four-wheel steering, or active stabilizers in order to design rollover controllers to achieve the desired vehicle behavior. In [4], an LQR-based controller with an estimation of the road bank via an active suspension was presented. Several authors designed a Unified Chassis Control system using a roll state estimator in order to prevent rollover [5] as well as both rollover prevention and lateral stability [6]. Related to the design of the rollover prevention controller, these can act on different elements of the vehicle. In [7], a differential braking rollover mitigation strategy for wheeled vehicles was presented. In [8], the C/GMRES method was used to solve an optimal rollover control problem in real time. In other studies [9], the actuator used to stabilize the roll angle was an active suspension.

On the other hand, the analysis and design of the controller focused on its implementation in embedded systems in vehicular applications is the subject of study in other works [10,11,12]. These controllers require all the main values of vehicle dynamics such as angular positions, accelerations, and angular rates [13,14,15,16]. Small size, fast processing time, and high precision are the main characteristics that these systems must have for vehicle applications. Furthermore, these systems must be low-cost in order to not increase the price of production of the vehicles. Previous works [17,18] analysed the integration of low-cost sensors in vehicles and the use of these values to estimate the roll angle with the use of different methods [19,20]. These research works demonstrated the feasibility of using these systems. However, these works also indicated that this type of low-cost kit could present relevant delays and noise that must be compensated in order to improve the performance of the system.

The phenomenon of time delay appears in many studies about complex systems control. Nowadays, vehicles have communication networks that connect their components, such as sensors, controllers, and actuators, which generate delays in sending data between these components. These delays can compromise the accuracy of the calculation of system variables or lengthen the actuation of the controllers, which may cause dangerous situations in real-time security applications. This has led to the design of the so-called Networked Control Systems (NCS) [21]. Consequently, several studies have focused on time delay compensation in state, input, or output signals. In [22], the transfer alignment time delay of the strapdown Inertial Navigation Systems (INS) was filtered using the H${}_{\infty }$ method. In [23], an H${}_{\infty }$ observer combined with NN for the estimation of the sideslip and roll angle with network-induced delays was designed. In other studies [24], a delay controller was designed for active vibration suppression systems. In [25], a controller was used to stabilize the lateral vehicle dynamics with a state delay of an active front steering system for electric vehicles. Other authors have designed an iterative learning system control with time-delay compensation for specific applications [26].

The novelty of this work is the development of an LMI-Based H${}_{\infty }$ output-feedback controller that compensates the input and output delays in a Roll Stability Control (RSC) system. This controller is designed for automotive applications based on low-cost systems such as those presented in [17,19,20]. This controller was implemented in a simulation environment using a validated vehicle model with TruckSim^®^. This article is organized as follows: In Section 2, the methodology is presented, including problem formulation, vehicle model characterization, and experiment definition. The simulation results and the calculation of the RMS values are presented in Section 3. Finally, the discussion of the results and the conclusion are presented in Section 4.

## 2. Methodology

This section describes the methodology adopted to develop an H${}_{\infty }$ output-feedback networked control system for vehicle roll stability with input and output delays.

**Figure 1:**
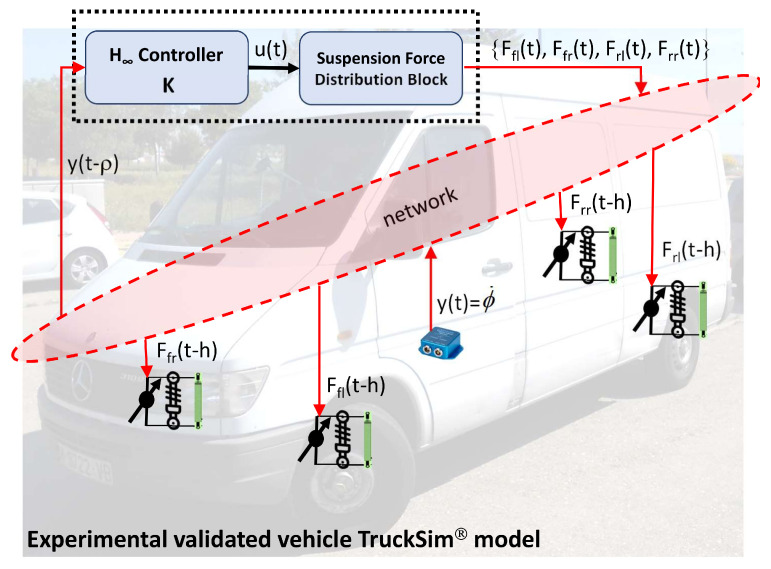
Controller Structure.

The structure of the proposed controller is shown in Figure 1. The roll rate $\dot{\phi }$ is the observable variable obtained from a gyro-oscilloscope that is already incorporated in current vehicles or from an IMU (Inertial Measurement Unit) located at the center of gravity of a real vehicle. In order for the price of current vehicles not to increase, this sensor can be a low-cost IMU that can give the same performance as high-performance sensors, as presented in [18,19,20].

**Figure 2:**
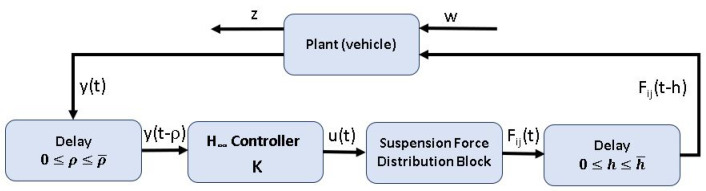
Control block diagram.

In this work, it is assumed that the plant measurements from sensors are sent to the controller through the network, and the control inputs are computed by the controller and transmitted back to the actuators through the network. Both the control input u(t) computed by the controller and the sensor measurements y(t) will experience delays. As depicted in Figure 2, a delay *h* is applied to the control input, and a delay ρ is applied to the data sensor. The lateral acceleration $a_{y}\left(t\right)$ obtained from an IMU and the road bank angle $\phi_{r}\left(t\right)$ are considered as external disturbances.

### 2.1. Vehicle Model Used in the Controller Design

**Figure 3:**
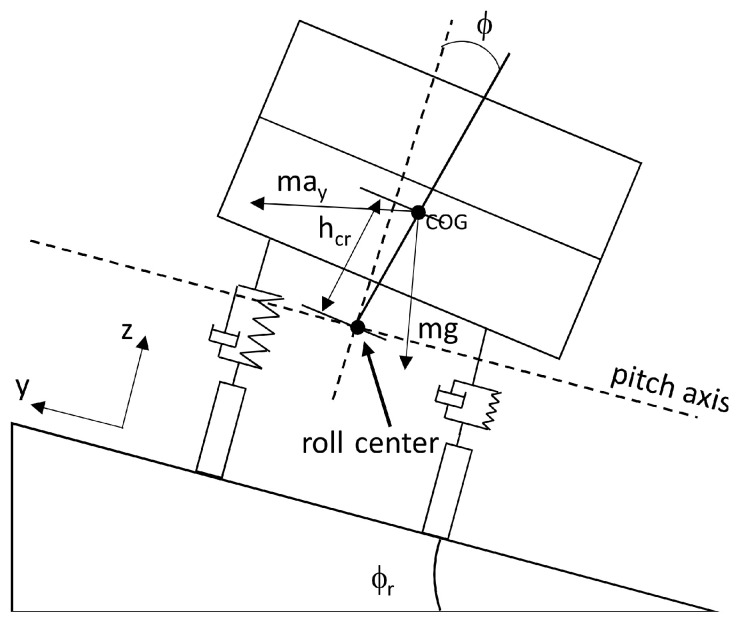
Vehicle model used in the controller design.

The vehicle model used in the H${}_{\infty }$ feedback-observer controller design is based on a Mercedes Benz Sprinter presented in [27]. This model describes the roll vehicle motion (see Figure 3), where ϕ is the roll angle and $a_{y}$ is the lateral acceleration.

The state space representation of the vehicle roll model used to define the controller presented in this work, taking into account the network delay, is as follows:

(1) $$\dot{x}\left(t\right)=\mathrm{Ax}\left(t\right)+B_{u}u\left(t-h\right)+B_{a_{y}}a_{y}\left(t\right)+B_{\phi_{r}}\phi_{r}\left(t\right)+B_{d}d_{s}\left(t\right)$$

(2) $$y\left(t\right)=C_{1}x\left(t\right)$$

(3) $$z\left(t\right)=C_{2}x\left(t\right)$$

where $x={\left[\phi ,\dot{\phi }\right]}^{T}$ is the state vector given by the roll angle ϕ and roll rate $\dot{\phi }$, $y=\dot{\phi }$ is the measured output, $u=M_{x}$ is the control input, $M_{x}$ is the anti-roll moment, z is the controlled vector, $a_{y}$ is the lateral acceleration, $\phi_{r}$ is the road bank angle, $d_{s}$ is the unknown vector disturbance, and

$$A=\left[\begin{array}{cc} 0 & 1\\ \frac{\left(m*g*h_{cr}\right)-K_{r}}{I_{xx}} & \frac{C_{r}}{I_{xx}} \end{array}\right];B_{u}=\left[\begin{array}{c} 0\\ \frac{1}{I_{xx}} \end{array}\right];\;B_{a_{y}}=\left[\begin{array}{c} 0\\ \frac{m\cdot h_{cr}}{I_{xx}} \end{array}\right]$$

$$B_{\phi_{r}}=\left[\begin{array}{c} 0\\ \frac{m\cdot h_{cr}\cdot g}{I_{xx}} \end{array}\right];\;B_{d}=\left[\begin{array}{c} 1\\ 1 \end{array}\right];$$

$$C_{1}=\left[\begin{array}{cc} 0 & 1 \end{array}\right]$$

(4) $$C_{2}=\left[\begin{array}{cc} 1 & 1 \end{array}\right]$$

Table 1 shows all the parameters of the vehicle model.

### 2.2. H${}_{\infty }$ Output-Feedback Control Design Considering Network Delays

For the design of the proposed H${}_{\infty }$ output-feedback controller, the control input given in Equation (1) is defined as follows:

(5) u(t)=Ky(t−ρ)

where **K** is the gain controller to be determined. In this case, the control input will depend on the observed measure, the roll rate. This measurement signal suffers a ρ delay because it is sent through the communication network. Thus, the system given in Equation (1) can be rewritten as:

(6) $$\begin{array}{c} \dot{x}\left(t\right)=\mathrm{Ax}\left(t\right)+B_{u}Ky\left(t-\tau \right)+B_{a_{y}}a_{y}\left(t\right)+B_{\phi_{r}}\phi_{r}\left(t\right)+B_{d}d_{s}\left(t\right) \end{array}$$

with τ = h + ρ.

Combining Equations (2) and (6), we get:

(7) $$\begin{array}{c} \dot{x}\left(t\right)=\mathrm{Ax}\left(t\right)+B_{u}{\mathrm{KC}}_{1}x\left(t-\tau \right)+B_{w}w\left(t\right) \end{array}$$

where $w\left(t\right)={\left[\begin{array}{ccc} a_{y}\left(t\right) & \phi_{r}\left(t\right) & d_{s}\left(t\right) \end{array}\right]}^{T}$ is the input disturbance and $B_{w}=\left[\begin{array}{ccc} B_{a_{y}} & B_{\phi_{r}} & B_{d} \end{array}\right]$.

The network transmission delays are bounded as 0 ≤ h ≤ $\bar{h}$ and 0 ≤ ρ ≤ $\bar{\rho }$, where $\bar{h}$ and $\bar{\rho }$ are the maximum delays for the input and output signals, respectively.

**Theorem** **1.** *For given scalars $\bar{h}$ > 0, $\bar{\phi }$ > 0, the networked system (7) is asymptotically stable with an H${}_{\infty }$ performance index γ if there exist a positive scalar γ, defined positive symmetric matrices**X*** *=* ***X*** *${}^{T}$* > ***0*** *,* ***H*** *=* ***H*** *${}^{T}$ >* ***0*** *,* ***Y*** *=* ***Y*** *${}^{T}$ >* ***0*** *,* ***Q*** *=* ***Q*** *${}^{T}$ >* ***0*** *, and any matrix* ***W*** *, which satisfy the following LMIs:*

(8) $$\;\begin{array}{c} \left[\begin{array}{ccccccc} {\mathrm{XA}}^{T}+\mathrm{AX}+B_{u}W+W^{T}{B_{u}}^{T} & 0 & B_{w} & -B_{u}Y & {\mathrm{XA}}^{T}+W^{T}B_{u}^{T} & {\mathrm{XC}}_{2}^{T} & W^{T}\\ {}* & -L & 0 & 0 & 0 & 0 & 0\\ {}* & * & -\gamma^{2}I & 0 & {B_{w}}^{T} & 0 & 0\\ {}* & * & * & \frac{-1}{\tau }Y & {\mathrm{YB}}_{u}^{T} & 0 & 0\\ {}** & * & * & \frac{-1}{\tau }Q & 0 & 0\\ {}** & * & * & * & -I & 0\\ {}** & * & * & * & * & -L \end{array}\right]≺0 \end{array}$$

(9) $$\begin{array}{c} \left[\begin{array}{cc} -2X+Q & -W^{T}\\ W & -Y \end{array}\right]≺0 \end{array}$$

**Proof.**  Considering $\bar{\tau }$ = $\bar{h}$+$\bar{\rho }$ and using the Newton–Leibniz formulas,

(10) $$\begin{array}{c} B_{u}{\mathrm{KC}}_{1}\int_{t-\tau }^{t}\dot{x}\left(s\right)ds=B_{u}{\mathrm{KC}}_{1}x\left(t\right)-B_{u}{\mathrm{KC}}_{1}x\left(t-\tau \right) \end{array}$$

(11) $$\begin{array}{c} B_{u}{\mathrm{KC}}_{1}x\left(t-\tau \right)=B_{u}{\mathrm{KC}}_{1}x\left(t\right)-B_{u}{\mathrm{KC}}_{1}\int_{t-\tau }^{t}\dot{x}\left(s\right)ds \end{array}$$

Equation (7) can be rewritten as follows:

(12) $$\begin{array}{c} \dot{x}=\mathrm{Ax}\left(t\right)+B_{u}{\mathrm{KC}}_{1}x\left(t\right)-B_{u}{\mathrm{KC}}_{1}\int_{t\;-\;\tau }^{t}\dot{x}\left(s\right)ds+B_{w}w\left(t\right) \end{array}$$

We then choose the following Lyapunov–Krasovskii functional candidate:

(13) $$\begin{array}{c} V\left(t\right)=V_{1}\left(t\right)+V_{2}\left(t\right)+V_{3}\left(t\right) \end{array}$$

with

(14) $$\begin{array}{c} V_{1}\left(t\right)=x^{T}\left(t\right)\mathrm{Px}\left(t\right) \end{array}$$

(15) $$\begin{array}{c} V_{2}\left(t\right)=\int_{t\;-\;\tau }^{t}{\dot{x}}^{T}\left(s\right){\left(C_{1}K\right)}^{T}{\mathrm{SKC}}_{1}x\left(s\right)ds \end{array}$$

(16) $$\begin{array}{c} V_{3}\left(t\right)=\int_{t\;-\;\tau }^{t}\left(s-\left(t-\tau \right)\right){\dot{x}}^{T}\left(s\right){\left({\mathrm{KC}}_{1}\right)}^{T}R\left({\mathrm{KC}}_{1}\right)\dot{x}\left(s\right)ds \end{array}$$

and **P** = **P**${}^{T}$ > 0, **R** = **R**${}^{T}$ > 0, **S** = **S**${}^{T}$ > 0.

Taking the derivative of *V*(*t*) with respect to *t* along the trajectory of system (7), we have the following equation:

(17) $$\begin{array}{c} {\dot{V}}_{1}\left(t\right)={\dot{x}}^{T}\left(t\right)Px\left(t\right)+x^{T}\left(t\right)P\dot{x}\left(t\right)=x^{T}\left(t\right)\left[{\left(A+B_{u}{\mathrm{KC}}_{1}\right)}^{T}P+P\left(A+B_{u}{\mathrm{KC}}_{1}\right)\right]x\left(t\right)\\ -2x{\left(t\right)}^{T}{\mathrm{PB}}_{u}{\mathrm{KC}}_{1}\int_{t-\tau }^{t}\dot{x}\left(s\right)ds+2x{\left(t\right)}^{T}PB_{w}w\left(t\right) \end{array}$$

(18) $$\begin{array}{c} {\dot{V}}_{2}\left(t\right)=x^{T}\left(t\right){\left(C_{1}K\right)}^{T}{\mathrm{SKC}}_{1}x\left(t\right)-x^{T}\left(t-\tau \right){\left(C_{1}K\right)}^{T}{\mathrm{SKC}}_{1}x\left(t-\tau \right) \end{array}$$

Taking into account the inequality

(19) $$\begin{array}{c} -\int_{t\;-\;\sigma }^{t}{\dot{x}}^{T}\left(s\right)T\dot{x}\left(s\right)ds\leq -\frac{1}{\sigma }{\left[\int_{t-\sigma }^{t}\dot{x}\left(s\right)ds\right]}^{T}T\left[\int_{t-\sigma }^{t}\dot{x}\left(s\right)ds\right] \end{array}$$

we obtain

(20) $$\begin{array}{c} {\dot{V}}_{3}\left(t\right)=x^{T}\left(t\right){\left(A+B_{u}{\mathrm{KC}}_{1}\right)}^{T}\tau {\left({\mathrm{KC}}_{1}\right)}^{T}R\left({\mathrm{KC}}_{1}\right)\left(A+B_{u}{\mathrm{KC}}_{1}\right)x\left(t\right)\\ -\left[\int_{t\;-\;\tau }^{t}{\dot{x}}^{T}\left(s\right)ds\right]{\left({\mathrm{KC}}_{1}\right)}^{T}B_{u}^{T}\tau {\left({\mathrm{KC}}_{1}\right)}^{T}R\left({\mathrm{KC}}_{1}\right)B_{u}\left({\mathrm{KC}}_{1}\right)\left[\int_{t\;-\;\tau }^{t}\dot{x}\left(s\right)ds\right]\\ +w^{T}\left(t\right){B_{w}}^{T}\tau {\left({\mathrm{KC}}_{1}\right)}^{T}R\left({\mathrm{KC}}_{1}\right)B_{w}w\left(t\right)-\frac{1}{\tau }{\left[\int_{t-\tau }^{t}\dot{x}\left(s\right)ds\right]}^{T}{\left({\mathrm{KC}}_{1}\right)}^{T}R\left({\mathrm{KC}}_{1}\right)\left[\int_{t-\tau }^{t}\dot{x}\left(s\right)ds\right] \end{array}$$

Furthermore, the closed-loop system defined by Equation (12) has an H${}_{\infty }$ performance under zero initial condition if the following inequality is satisfied:

(21) $$\begin{array}{c} \|z^{T}{z\|}_{2}<\gamma \left\|w^{T}w\right\| \end{array}$$

Then, system (12) is asymptotically stable and has an H${}_{\infty }$ performance if the following inequality is satisfied:

(22) $$\begin{array}{c} \dot{V}\left(t\right)+z{\left(t\right)}^{T}z\left(t\right)-\gamma^{2}w{\left(t\right)}^{T}w\left(t\right)<0 \end{array}$$

(23) $$\begin{array}{c} x^{T}\left(t\right)\left[{\left(A+B_{u}{\mathrm{KC}}_{1}\right)}^{T}P+P\left(A+B_{u}{\mathrm{KC}}_{1}\right)\right]x\left(t\right)-2x{\left(t\right)}^{T}{\mathrm{PB}}_{u}{\mathrm{KC}}_{1}\int_{t-\tau }^{t}\dot{x}\left(s\right)ds\\ +2x{\left(t\right)}^{T}PB_{w}w\left(t\right)-x^{T}\left(t\right){\left(C_{1}K\right)}^{T}{\mathrm{SKC}}_{1}x\left(t\right)-x^{T}\left(t-\tau \right){\left(C_{1}K\right)}^{T}{\mathrm{SKC}}_{1}x\left(t-\tau \right)\\ +x^{T}\left(t\right){\left(A+B_{u}{\mathrm{KC}}_{1}\right)}^{T}\tau {\left({\mathrm{KC}}_{1}\right)}^{T}R\left({\mathrm{KC}}_{1}\right)\left(A+B_{u}{\mathrm{KC}}_{1}\right)x\left(t\right)\\ -\left[\int_{t\;-\;\tau }^{t}{\dot{x}}^{T}\left(s\right)ds\right]{\left({\mathrm{KC}}_{1}\right)}^{T}B_{u}^{T}\tau {\left({\mathrm{KC}}_{1}\right)}^{T}R\left({\mathrm{KC}}_{1}\right)B_{u}\left({\mathrm{KC}}_{1}\right)\left[\int_{t\;-\;\tau }^{t}\dot{x}\left(s\right)ds\right]\\ +w^{T}\left(t\right){B_{w}}^{T}\tau {\left({\mathrm{KC}}_{1}\right)}^{T}R\left({\mathrm{KC}}_{1}\right)B_{w}w\left(t\right)-\frac{1}{\tau }{\left[\int_{t-\tau }^{t}\dot{x}\left(s\right)ds\right]}^{T}{\left({\mathrm{KC}}_{1}\right)}^{T}R({\mathrm{KC}}_{1}\left[\int_{t-\tau }^{t}\dot{x}\left(s\right)ds\right]\\ +{\left(C_{2}x\left(t\right)\right)}^{T}C_{2}x\left(t\right)-\gamma^{2}w{\left(t\right)}^{T}w\left(t\right)<0 \end{array}$$

Now, let a new state vector be defined as follows:

(24) $$\begin{array}{c} \psi^{T}\left(t\right)=\left[x^{T}\left(t\right),x^{T}\left(t-\tau \right){\left(C_{1}K\right)}^{T},w^{T}\left(t\right),\int_{t-\tau }^{t}{\dot{x}}^{T}\left(s\right)ds{\left({\mathrm{KC}}_{1}\right)}^{T}\right] \end{array}$$

Then,

(25) $$\begin{array}{c} \psi {\left(t\right)}^{T}\Sigma_{0}\psi \left(t\right)≺0 \end{array}$$

where

(26) $$\begin{array}{c} \Sigma_{0}=\left[\begin{array}{cccc} {▵}_{11} & 0 & PB_{w} & -PB_{u}\\ {}* & -S & 0 & 0\\ {}* & * & -\gamma^{2}I & 0\\ {}* & * & * & \frac{-1}{\tau }R \end{array}\right]+\left[\begin{array}{c} {{▵}_{1}}^{T}\\ 0\\ {B_{w}}^{T}\\ {B_{u}}^{T} \end{array}\right]\left(\tau {\left({\mathrm{KC}}_{1}\right)}^{T}R\left({\mathrm{KC}}_{1}\right)\right){\left[\begin{array}{c} {▵}_{1}\\ 0\\ B_{w}\\ B_{u} \end{array}\right]}^{T} \end{array}$$

and

(27) $$\begin{array}{c} {▵}_{11}={\left(A+B_{u}{\mathrm{KC}}_{1}\right)}^{T}P+P\left(A+B_{u}{\mathrm{KC}}_{1}\right)+{C_{2}}^{T}C_{2}+{\left(C_{1}K\right)}^{T}{\mathrm{SC}}_{1}K \end{array}$$

(28) $$\begin{array}{c} {▵}_{1}=A+B_{u}{\mathrm{KC}}_{1} \end{array}$$

Equation (25) can then be rewritten as follows:

(29) $$\begin{array}{c} \Sigma_{0}≺0 \end{array}$$

We define the matrix η with

(30) $$\begin{array}{c} \eta =\left[\begin{array}{cccc} P^{-1} & 0 & 0 & 0\\ 0 & S^{-1} & 0 & 0\\ 0 & 0 & I & 0\\ 0 & 0 & 0 & R^{-1} \end{array}\right] \end{array}$$

Premultiplying and postmultiplying in Equation (26) by $\eta^{T}$ and η

(31) $$\begin{array}{c} \left[\begin{array}{cccc} P^{-1} & 0 & 0 & 0\\ 0 & S^{-1} & 0 & 0\\ 0 & 0 & I & 0\\ 0 & 0 & 0 & R^{-1} \end{array}\right]\left[\begin{array}{cccc} {▵}_{11} & 0 & PB_{w} & -PB_{u}\\ {}* & -S & 0 & 0\\ {}* & * & -\gamma^{2}I & 0\\ {}* & * & * & \frac{-1}{\tau }R \end{array}\right]\left[\begin{array}{cccc} P^{-1} & 0 & 0 & 0\\ 0 & S^{-1} & 0 & 0\\ 0 & 0 & I & 0\\ 0 & 0 & 0 & R^{-1} \end{array}\right]+\\ \left[\begin{array}{cccc} P^{-1} & 0 & 0 & 0\\ 0 & S^{-1} & 0 & 0\\ 0 & 0 & I & 0\\ 0 & 0 & 0 & R^{-1} \end{array}\right]\left[\begin{array}{c} {{▵}_{1}}^{T}\\ 0\\ {B_{w}}^{T}\\ {B_{u}}^{T} \end{array}\right]\left(\tau {\left({\mathrm{KC}}_{1}\right)}^{T}R\left({\mathrm{KC}}_{1}\right)\right){\left[\begin{array}{c} {▵}_{1}\\ 0\\ B_{w}\\ B_{u} \end{array}\right]}^{T}\left[\begin{array}{cccc} P^{-1} & 0 & 0 & 0\\ 0 & S^{-1} & 0 & 0\\ 0 & 0 & I & 0\\ 0 & 0 & 0 & R^{-1} \end{array}\right]=\\ \left[\begin{array}{cccc} {\hat{▵}}_{11} & 0 & B_{w} & -B_{u}R^{-1}\\ {}* & -S^{-1} & 0 & 0\\ {}* & * & -\gamma^{2}I & 0\\ {}* & * & * & \frac{-1}{\tau }R^{-1} \end{array}\right]+\left[\begin{array}{c} {{\hat{▵}}_{1}}^{T}\\ 0\\ {B_{w}}^{T}\\ {R^{-1}B_{u}}^{T} \end{array}\right]\left(\tau {\left({\mathrm{KC}}_{1}\right)}^{T}R\left({\mathrm{KC}}_{1}\right)\right){\left[\begin{array}{c} {\hat{▵}}_{1}\\ 0\\ B_{w}\\ B_{u}R^{-1} \end{array}\right]}^{T} \end{array}$$

with

(32) $$\begin{array}{c} {\hat{▵}}_{11}=P^{-1}{\left(A+B_{u}{\mathrm{KC}}_{1}\right)}^{T}+\left(A+B_{u}{\mathrm{KC}}_{1}\right)P^{-1}+{\left(C_{2}P^{-1}\right)}^{T}\left(C_{2}P^{-1}\right)+{\left(C_{1}{\mathrm{KP}}^{-1}\right)}^{T}S\left(C_{1}{\mathrm{KP}}^{-1}\right) \end{array}$$

(33) $$\begin{array}{c} {\hat{▵}}_{1}=P^{-1}\left(A+B_{u}{\mathrm{KC}}_{1}\right) \end{array}$$

We define the matrices **W**, **X**, **Y**, and **L** as:

(34) $$\begin{array}{c} W={\mathrm{KC}}_{1}P^{-1},X=P^{-1},Y=R^{-1},L=S^{-1} \end{array}$$

and apply the change of variable

(35) $$\begin{array}{c} \left[\begin{array}{cccc} {\bar{▵}}_{11} & 0 & B_{w} & -B_{u}Y\\ {}* & -L & 0 & 0\\ {}* & * & -\gamma^{2}I & 0\\ {}* & * & * & \frac{-1}{\tau }Y \end{array}\right]+\left[\begin{array}{c} {{\hat{▵}}_{1}}^{T}\\ 0\\ {B_{w}}^{T}\\ {YB_{u}}^{T} \end{array}\right]\left(\tau {\left({\mathrm{KC}}_{1}\right)}^{T}Y^{-1}\left({\mathrm{KC}}_{1}\right)\right){\left[\begin{array}{c} {\hat{▵}}_{1}\\ 0\\ B_{w}\\ B_{u}Y \end{array}\right]}^{T} \end{array}$$

(36) $$\begin{array}{c} {\bar{▵}}_{11}={\mathrm{XA}}^{T}+\mathrm{AX}+W^{T}B_{u}^{T}+\mathrm{BuW}+XC_{2}^{T}C_{2}X+WL^{-1}W \end{array}$$

(37) $$\begin{array}{c} {\bar{▵}}_{1}=P^{-1}\left(A+B_{u}{\mathrm{KC}}_{1}\right) \end{array}$$

If the following inequality is satisfied

(38) $$\begin{array}{c} \left[\begin{array}{cc} -2X+Q & W^{T}\\ W & -Y \end{array}\right]≺0 \end{array}$$

with **Q** = **Q**${}^{T}$ > 0, **X** = **X**${}^{T}$ > 0, and the Schur complement is applied, we get the following:

(39) $$\begin{array}{c} -2X+Q+W^{T}Y^{-1}W≺0 \end{array}$$

Taking **W** = **K**$C_{1}$**X** and the following inequality:

(40) $$\left(X-Q\right)Q^{-1}\left(X-Q\right)=XQ^{-1}X-2X+Q≻0$$

it is guaranteed that (39) implies

(41) $$\begin{array}{c} {\mathrm{KC}}_{1}^{T}Y^{-1}{\mathrm{KC}}_{1}≺Q^{-1} \end{array}$$

Using (41), Equation (35) can be transformed into:

(42) $$\begin{array}{c} \left[\begin{array}{cccc} {\bar{▵}}_{11} & 0 & B_{w} & -B_{u}Y\\ {}* & -L & 0 & 0\\ {}* & * & -\gamma^{2}I & 0\\ {}* & * & * & \frac{-1}{\tau }Y \end{array}\right]+\left[\begin{array}{c} {{\hat{▵}}_{1}}^{T}\\ 0\\ {B_{w}}^{T}\\ {YB_{u}}^{T} \end{array}\right]\left(\tau Q^{-1}\right){\left[\begin{array}{c} {\hat{▵}}_{1}\\ 0\\ B_{w}\\ B_{u}Y \end{array}\right]}^{T} \end{array}$$

After applying the Schur complement,

(43) $$\begin{array}{c} \left[\begin{array}{ccccc} {\bar{▵}}_{11} & 0 & B_{w} & -B_{u}Y & {\mathrm{XA}}^{T}+W^{T}B_{u}^{T}\\ {}* & -L & 0 & 0 & 0\\ {}* & * & -\gamma^{2}I & 0 & {B_{w}}^{T}\\ {}* & * & * & \frac{-1}{\tau }Y & {{\mathrm{YB}}_{u}}^{T}\\ {}* & * & * & * & \frac{-1}{\tau }Q \end{array}\right] \end{array}$$

After applying the Schur complement again, it is understood that for any constant time delay τ < $\bar{\tau }$, the system (1) is stable and that there exist positive and symmetric matrices **X**, **L**, **Y**, and **Q** such that the following LMIs are feasible:

(44) $$\;\begin{array}{c} \left[\begin{array}{ccccccc} {\mathrm{XA}}^{T}+\mathrm{AX}+B_{u}W+W^{T}{B_{u}}^{T} & 0 & B_{w} & -B_{u}Y & {\mathrm{XA}}^{T}+W^{T}B_{u}^{T} & {\mathrm{XC}}_{2}^{T} & W^{T}\\ {}* & -L & 0 & 0 & 0 & 0 & 0\\ {}* & * & -\gamma^{2}I & 0 & {B_{w}}^{T} & 0 & 0\\ {}* & * & * & \frac{-1}{\tau }Y & {\mathrm{YB}}_{u}^{T} & 0 & 0\\ {}* & * & * & * & \frac{-1}{\tau }Q & 0 & 0\\ {}* & * & * & * & * & -I & 0\\ {}* & * & * & * & * & * & -L \end{array}\right]≺0 \end{array}$$

(45) $$\begin{array}{c} \left[\begin{array}{cc} -2X+Q & -W^{T}\\ W & -Y \end{array}\right]≺0 \end{array}$$

From Equations (22)–(25), we obtain

(46) $$\begin{array}{c} \dot{V}+z^{T}z-\gamma^{2}w^{T}w<\psi^{T}\Sigma_{1}\psi \end{array}$$

Under a zero initial condition, integrating the inequality given in Equation (46) from t = 0 to t = *∞* yields Equation (21) for all non-zero external disturbances, then an H${}_{\infty }$ performance is guaranteed. Similarly, if w(t) = 0, it follows that $\dot{V}$ < 0, which means that the system is asymptotically stable.

The proof is complete. □

### 2.3. H${}_{\infty }$ Output-Feedback Controller Design without Considering Network Delays

In order to compare the performance of the proposed controller in Section 2.2, the results were compared with the results obtained using an H${}_{\infty }$ controller that did not consider the delay in its designs. The system (1) can be redesigned as follows:

(47) $$\begin{array}{c} \dot{x}\left(t\right)=\mathrm{Ax}\left(t\right)+B_{u}u\left(t\right)+B_{a_{y}}a_{y}\left(t\right)+B_{\phi_{r}}\phi_{r}\left(t\right)+B_{d}d_{s}\left(t\right) \end{array}$$

(48) $$\begin{array}{c} y\left(t\right)=C_{1}x\left(t\right) \end{array}$$

(49) $$\begin{array}{c} z\left(t\right)=C_{2}x\left(t\right) \end{array}$$

The Lyapunov–Krasovskii functional candidate in this case is:

(50) $$\begin{array}{c} V\left(t\right)=V_{1}\left(t\right) \end{array}$$

with

(51) $$\begin{array}{c} V_{1}\left(t\right)=x^{T}\left(t\right)\mathrm{Px}\left(t\right) \end{array}$$

Developing the system in the same way as in Section 2.2, the system (47) is stable with an H${}_{\infty }$ performance, and there exists a positive and symmetric matrix **X** such that the following LMI is feasible:

(52) $$\begin{array}{c} \left[\begin{array}{ccc} {\mathrm{XA}}^{T}+\mathrm{AX}+B_{u}W+W^{T}B_{u}^{T} & B_{w} & {\mathrm{XC}}_{2}^{T}\\ {}* & -\gamma^{2}I & 0\\ {}* & * & -I \end{array}\right]≺0 \end{array}$$

### 2.4. Suspension Force Distribution Block

**Figure 4:**
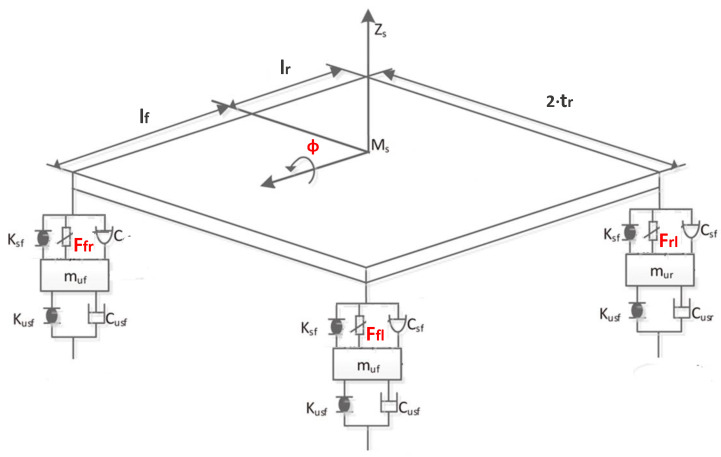
Distribution of active forces.

The controller selected for the roll angle stabilization and performance verification of the developed system is based on an active suspension [28]. The considered vehicle has four active suspension systems, where the force on each shock absorber can be individually regulated. The forces to be exerted on each actuator mounted on the suspension are determined by the following expressions (see Figure 4):

(53) $$\begin{array}{c} F_{fl}=\mathrm{Front}\;\mathrm{left}\;\mathrm{suspension}\;\mathrm{vertical}\;\mathrm{force}=0.5\cdot u\left(t-h\right)\cdot \frac{l_{r}}{t_{f}\cdot l} \end{array}$$

(54) $$\begin{array}{c} F_{fr}=\mathrm{Front}\;\mathrm{right}\;\mathrm{suspension}\;\mathrm{vertical}\;\mathrm{force}=-0.5\cdot u\left(t-h\right)\cdot \frac{l_{r}}{t_{f}\cdot l} \end{array}$$

(55) $$\begin{array}{c} F_{rl}=\mathrm{Rear}\;\mathrm{left}\;\mathrm{suspension}\;\mathrm{vertical}\;\mathrm{force}=0.5\cdot u\left(t-h\right)\cdot \frac{l_{f}}{t_{r}\cdot l} \end{array}$$

(56) $$\begin{array}{c} F_{rr}=\mathrm{Rear}\;\mathrm{right}\;\mathrm{suspension}\;\mathrm{vertical}\;\mathrm{force}=-0.5\cdot u\left(t-h\right)\cdot \frac{l_{f}}{t_{r}\cdot l} \end{array}$$

All the values used to calculate the vertical forces are defined in Table 1.

**Figure 5:**
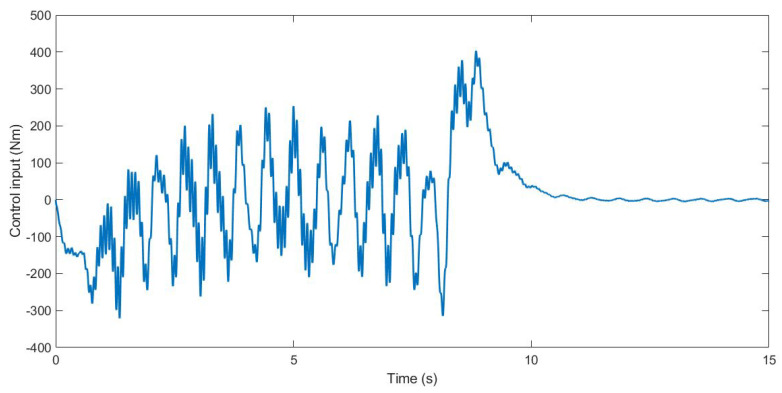
Control input *u* for Test 1.

**Figure 6:**
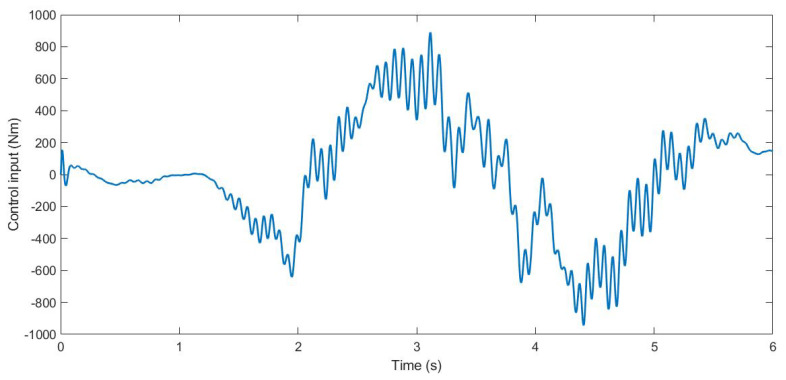
Control input *u* for Test 2.

**Figure 7:**
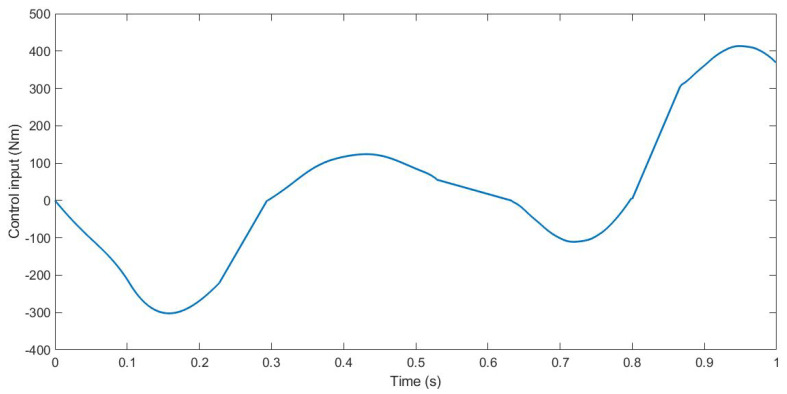
Control input *u* for Test 3.

During the simulation, the forces applied to the suspension change according to the value *u*. The control input *u* is the anti-roll moment that must be provided to the vehicle so that it maintains its stability. Figure 5, Figure 6 and Figure 7 show the control input *u* for the three tests. With this information, in the next section, the PSD value is calculated to evaluate the chattering phenomenon in order to avoid damages in the actuator that could derive from high-frequency changes.

## 3. Results

The control gain, **K**, of the proposed controller that considered the delays in the communication network was obtained by solving the following minimization problem:

(57) $$\begin{array}{cc} \min & \gamma^{2}\\ \mathrm{subject}\mathrm{to} & X=X^{T}≻0,H=H^{T}≻0,Y=Y^{T}≻0,\\ \; & Q=Q^{T}≻0 \end{array}$$

and Equations (8) and (9).

The values of the matrices, obtained by solving the minimization problem using the MATLAB^®^ LMI Toolbox^TM^, are as follows:

(58) $$\begin{array}{c} X=1\cdot 10^{-12}\left[\begin{array}{cc} 0.0043 & 0.0567\\ 0.0567 & 0.7181 \end{array}\right] \end{array}$$

(59) $$\begin{array}{c} W=1\cdot 10^{-10}\left[\begin{array}{cc} -0.1125 & -0.5985 \end{array}\right] \end{array}$$

(60) $$\begin{array}{c} K=W\cdot inv(C_{1}X)=-84.06 \end{array}$$

(61) $$\begin{array}{c} \gamma^{2}=0.09932 \end{array}$$

The maximum delay that allowed for the minimization problem to be solved is:

(62) $$\bar{\tau }=h+\rho =0.1\;s$$

For the controller that did not consider the delay in its design, the control gain, **K**, was calculated as follows:

(63) $$\begin{array}{cc} \min & \gamma^{2}\\ \mathrm{subject}\mathrm{to} & X=X^{T}≻0, \end{array}$$

and Equation (52).

The values of the matrices obtained by solving the minimization problem are:

(64) $$\begin{array}{c} X=\left[\begin{array}{cc} 41.5153 & -1.1369\\ -1.1369 & 5.5769 \end{array}\right] \end{array}$$

(65) $$\begin{array}{c} W=1\cdot 10^{7}\left[\begin{array}{cc} 3.1153 & -0.1178 \end{array}\right] \end{array}$$

(66) $$\begin{array}{c} K=W\cdot inv\left(C_{1}X\right)=-1.29\times 10^{6} \end{array}$$

(67) $$\begin{array}{c} \gamma^{2}=0.09932 \end{array}$$

### 3.1. Experiment Specification

**Figure 8:**
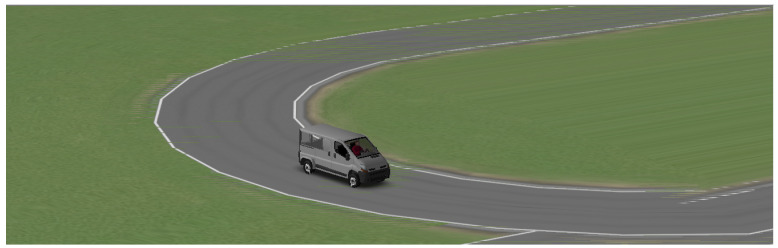
Test 1 and Test 3: Roundabout with a radius of 22 m.

**Figure 9:**
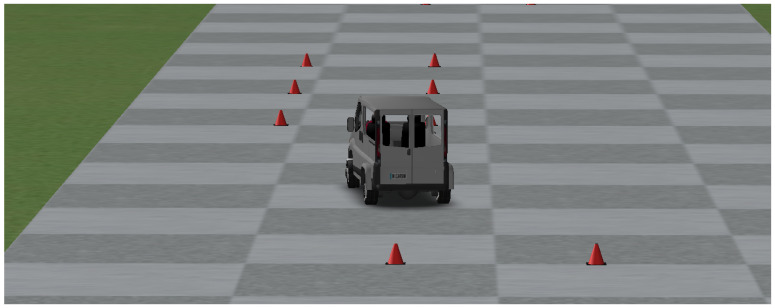
Test 2: Double lane change.

Several tests were performed to measure the performance of the controller through the TruckSim^®^ software and MATLAB-Simulink^®^. As mentioned in Section 2.1, a Mercedes Benz Sprinter model was used to carry out the simulation. This model was experimentally validated in [4,29]. The experiments selected for the simulated environment were a recreation of the experiments performed in previous works with a real vehicle [17,18,19,20]:

- Test 1: A roundabout with a radius of 22 m, at a constant speed of 30 km/h on dry pavement (see Figure 8).
- Test 2: A double lane change at a constant speed of 100 km/h on dry pavement (see Figure 9).
- Test 3: The same roundabout in Test 1, at a constant speed of 120 km/h, in order to evaluate the performance in a more severe test (see Figure 8).

For each test, three different scenarios were considered:

- A simulated scenario, with a delay of τ applied using the controller proposed in Section 2.2. This simulated scenario will appear in blue with the label *“H${}_{\infty }$ Controller (Proposed in this research)”* in all the figures of Section 3.
- A simulated scenario without a control system. This simulated scenario will appear in red with the label *“System Without Control”* in all the figures of Section 3.
- A simulated scenario, with a delay of τ applied using an H${}_{\infty }$ controller that did not take into account the delay in its design. This simulated scenario will appear in yellow with the label *H${}_{\infty }$ Controller (Without delay in its design)”* in all the figures of Section 3.

In all the scenarios, the roll angle of the vehicle was measured. To quantify the performance of the controller, the RMS and maximum errors of the roll angle were calculated. The roll angle error was established using an angle of 0° as the ground truth, that is, there is no roll in the vehicle. In addition to the two variables mentioned above, the normalized load transfer (NLT) in both axles was calculated. This variable is one of the most direct and accurate measures for evaluating RSC performance. It guarantees that the vehicle will not roll over when the normalized load transfer for both axles is below the value ± 1. The normalized load transfer can be calculated as [30]:

(68) $$\begin{array}{c} NLT_{f}=\frac{▵F_{zf}}{F_{zf}},NLT_{r}=\frac{▵F_{zr}}{F_{zr}} \end{array}$$

where $F_{zf}$ and $F_{zr}$ are the total load on the front and rear axle, respectively:

(69) $$\begin{array}{c} F_{zf}=\frac{l_{r}}{l_{f}+l_{r}}\mathrm{mg};\;F_{zr}=\frac{l_{f}}{l_{f}+l_{r}}\mathrm{mg}, \end{array}$$

$▵F_{zr}$ and $▵F_{zf}$ are the lateral load transfer values for the front and rear axle, respectively:

(70) $$\begin{array}{c} ▵F_{zf}=\frac{K_{r}\phi_{f}}{t_{r}};\;▵F_{zr}=\frac{K_{r}\phi_{r}}{t_{r}} \end{array}$$

The delay τ used during the simulations is the maximum delay that allows for the LMI (8) to have a solution (τ = 0.1 s). For the simulations, the delays considered were *h* = 0.05 s and ρ = 0.05 s.

The simulation results for the three tests defined in Section 3.1 are shown below.

#### 3.1.1. Test 1: Roundabout

**Figure 10:**
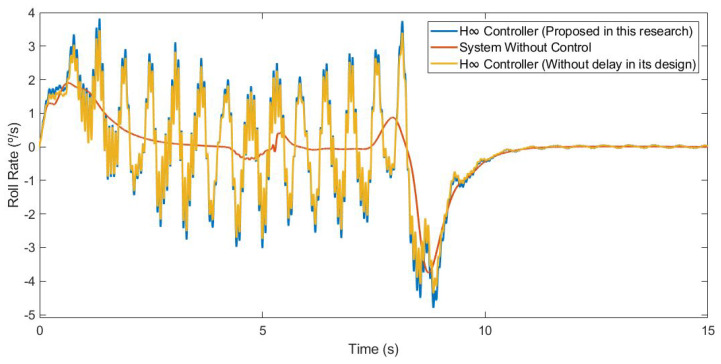
Roll rate for test 1: Roundabout at 30 km/h.

**Figure 11:**
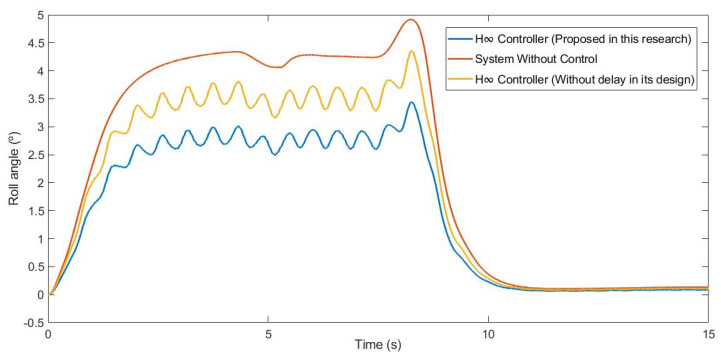
Roll angle for test 1: Roundabout at 30 km/h.

This first test was carried out by simulating the vehicle negotiating a roundabout with a radius of 22 m, at a constant speed of 30 km/h, on dry pavement. Figure 10 and Figure 11 show the roll rate and roll angle obtained in the three scenarios presented in Section 3.1. In blue is a simulated scenario with a delay of τ = 0.1 s applied using the controller proposed. In red is a simulated scenario without a control system. In yellow is a simulated scenario with a delay of τ = 0.1 s applied using an H${}_{\infty }$ controller that did not take into account the delay in its design. In Figure 11, it can be seen that the values obtained with the controller are lower than those of the systems it was compared with. In Table 2, the values are given. Results show that the vehicle roll angle decreases when using the controller proposed in this work as compared to a system with a standard H${}_{\infty }$ controller. Concerning the RMS and maximum errors, the difference between the proposed controller and the one that did not consider delay in its design is 0.55° and 0.91°, respectively. The differences between the proposed system and the system without a control are higher: 1.09° for the RMS error and 1.47° for the maximum error.

**Figure 12:**
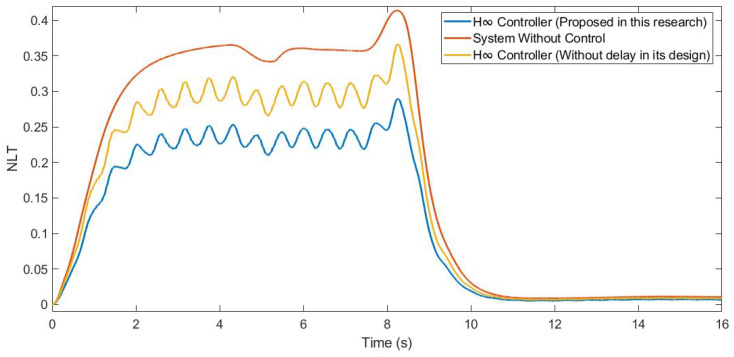
NLT of front axle for test 1: Roundabout at 30 km/h.

**Figure 13:**
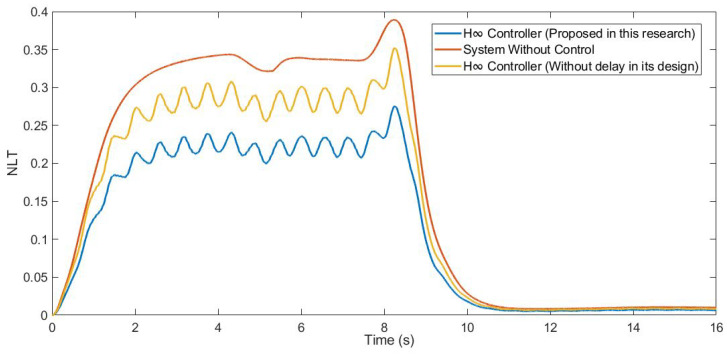
NLT of rear axle for test 1: Roundabout at 30 km/h.

Furthermore, the NLT value of the vehicle was calculated (see Figure 12 and Figure 13). As can be seen in Table 3, the NLT value follows the same trend as in the previous simulation. The difference between the controller designed in this research and the controller that did not consider the delay is 0.07 for both axles. Regarding the uncontrolled system, the difference is 0.11 for the front axle and 0.1 for the rear axle.

**Figure 14:**
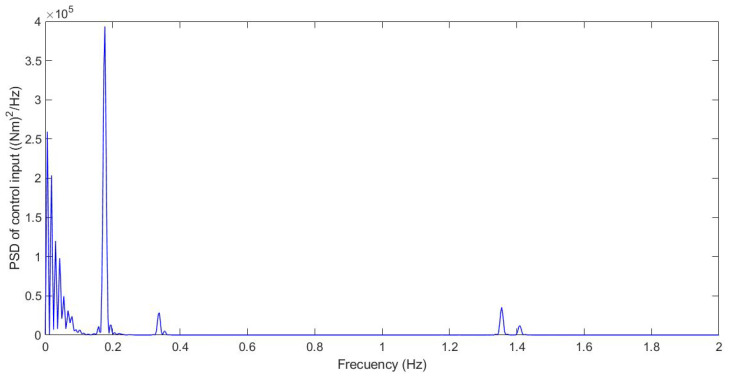
Frequency response for test 1: Roundabout at 30 km/h.

Additionally, the chattering phenomenon was analysed; this was caused by high-frequency changes in the control signal. This chatter may damage the actuator and compromise the control performance. Figure 14 shows the power spectral density (PSD) for the control input.

#### 3.1.2. Test 2: Double Lane Change

**Figure 15:**
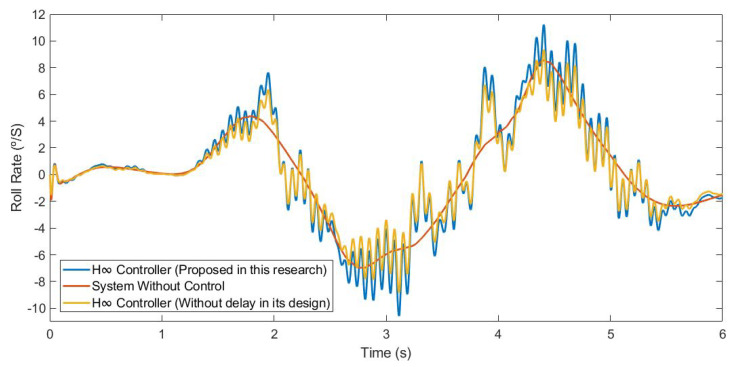
Roll rate for test 2: Double lane change at 100 km/h.

**Figure 16:**
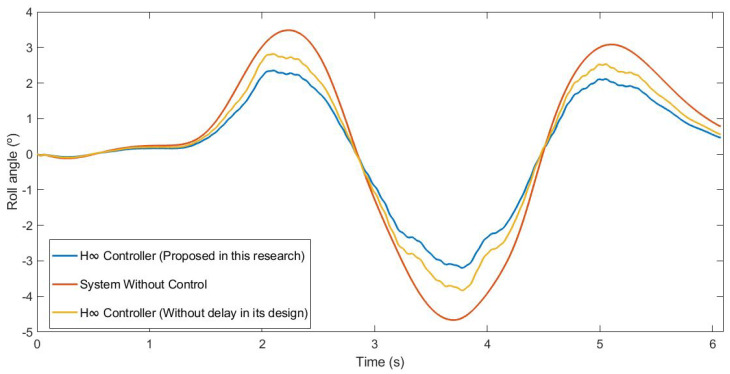
Roll angle for test 2: Double lane change at 100 km/h.

This second test was carried out by simulating the vehicle doing a slalom manoeuvre at 100 km/h on dry pavement. Figure 15 and Figure 16 show the roll rate and roll angle obtained in the three scenarios presented in Section 3.1. In blue is a simulated scenario with a delay of τ = 0.1 s applied using the controller proposed. In red is a simulated scenario without a control system. In yellow is a simulated scenario with a delay of τ = 0.1 s applied using an H${}_{\infty }$ controller that did not take into account the delay in its design. In Figure 16, it can be seen that the values obtained with the controller are lower than those of the systems it was compared with. To quantify the performance of the controller, the RMS and maximum errors of the roll angle were calculated. In Table 4, the values are given. Results show that the vehicle roll angle decreases when using the controller proposed in this work as compared to a system with a standard H${}_{\infty }$ controller. Concerning the RMS and maximum errors, the difference between the proposed controller and the one that did not consider the delay in its design is 0.31° and 0.46°, respectively. The differences between the proposed system and the uncontrolled system are higher: 0.8° for the RMS error and 1.13° for the maximum error.

**Figure 17:**
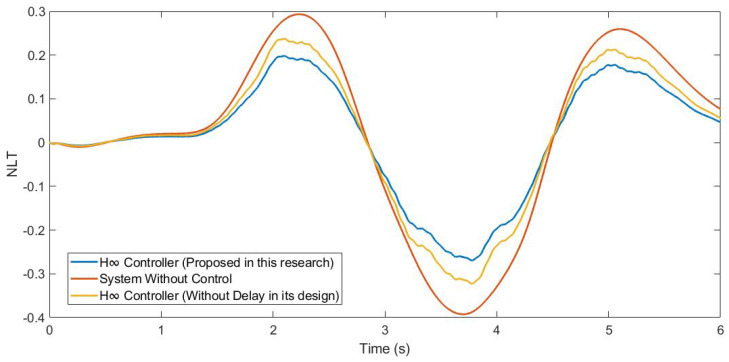
NLT of front axle for test 2: Double lane change at 100 km/h.

**Figure 18:**
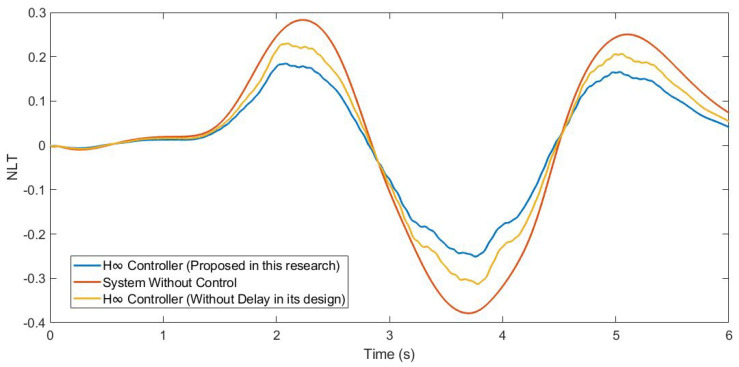
NLT of rear axle for test 2: Double lane change at 100 km/h.

Moreover, the NLT value of the vehicle was calculated (see Figure 17 and Figure 18). As can be seen in Table 5, the NLT value follows the same trend as that in the previous simulation. The difference between the controller designed in this research and the controller that did not consider the delay is 0.01 for both angles. Regarding the uncontrolled system, the difference is 0.1 for the front axle and 0.09 for the rear axle.

**Figure 19:**
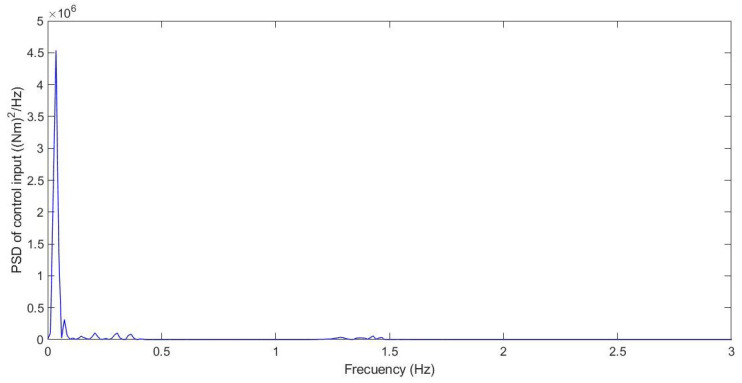
Frequency response for test 2: Double lane change at 100 km/h.

Additionally, the chattering phenomenon was analysed; this was caused by high-frequency changes in the control signal. This chatter may damage the actuator and compromise the control performance. Figure 19 shows the power spectral density (PSD) for the control input.

#### 3.1.3. Test 3: Roundabout

**Figure 20:**
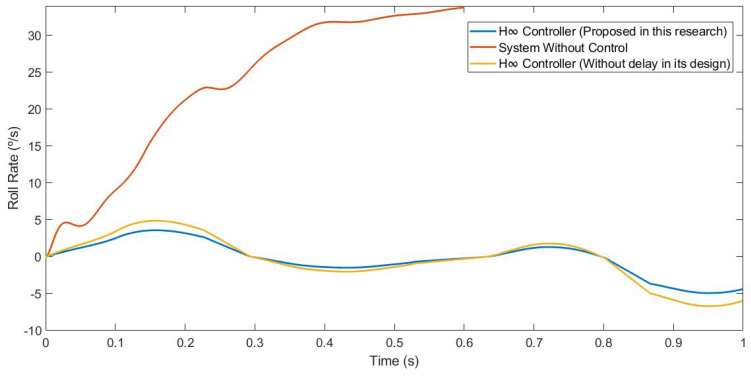
Roll rate for test 3: Roundabout at 120 km/h.

**Figure 21:**
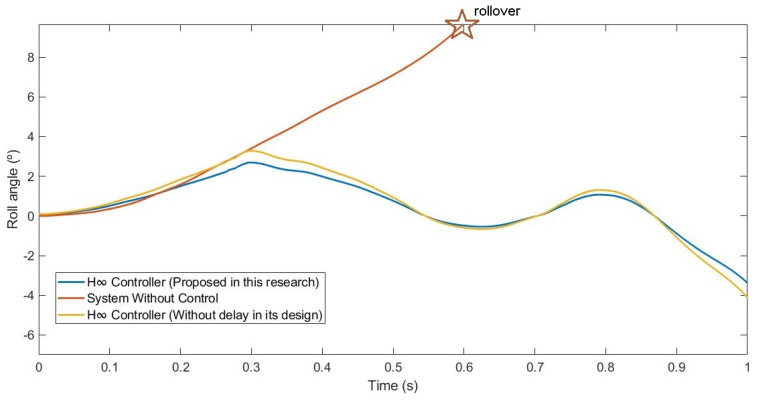
Roll angle for test 3: Roundabout at 120 km/h.

**Figure 22:**
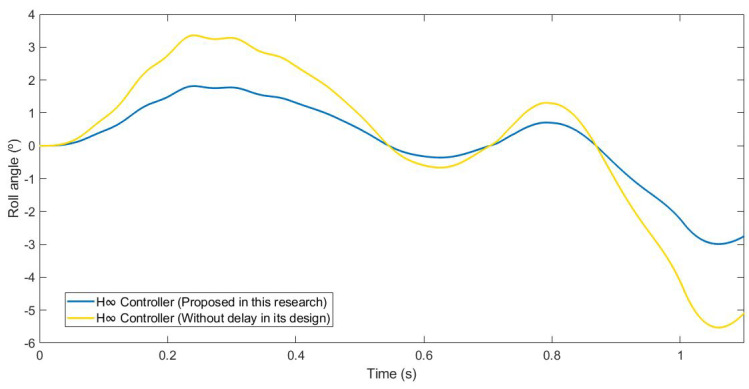
Zoom of the roll angle for test 3: Roundabout at 120 km/h.

This last test was carried out by simulating the vehicle negotiating a roundabout with a radius of 22 m, at a constant speed of 120 km/h, on dry pavement. This test was considered in order to evaluate the performance in a more severe test. Figure 20 and Figure 21 show the roll rate and roll angle obtained in the three scenarios presented in Section 3.1. In blue is a simulated scenario with a delay of τ = 0.1 s applied using the controller proposed. In red is a simulated scenario without a control system. In yellow is a simulated scenario with a delay of τ = 0.1 s applied using an H${}_{\infty }$ controller that did not take into account the delay in its design. In this test, the vehicle did not have any controller rollover, unlike in the controlled systems where the maximum angle was about 6°. In Figure 22, it can be seen that the values obtained with the controller proposed in this research are lower than the values obtained in the system with an H${}_{\infty }$ the controller. To quantify the performance of the controller, the RMS and maximum errors of the roll angle were calculated. In Table 6, the values are given. Results show that the vehicle roll angle decreases when using the controller proposed in this work as compared to a system with a standard H${}_{\infty }$ controller. Concerning the RMS and maximum errors, the difference between the proposed controller and the one that did not consider the delay in its design is 0.68° and 1.55°, respectively. The differences between the proposed system and the uncontrolled system are higher: 1.96° for the RMS error and 1.55° for the maximum error. Note that in the scenario without a controller, this should be considered a rollover.

**Figure 23:**
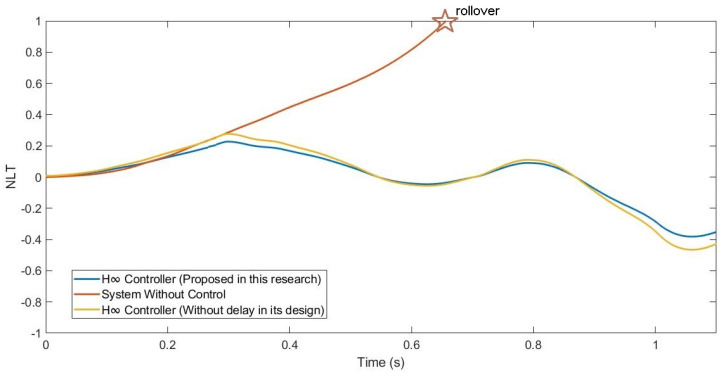
NLT of front axle for test 3: Roundabout at 120 km/h.

**Figure 24:**
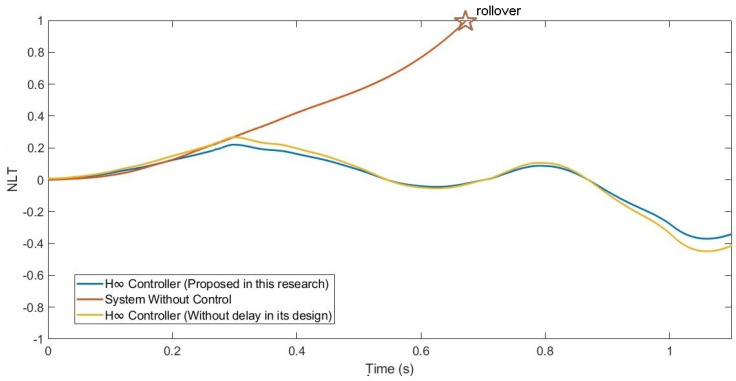
NLT of rear axle for test 3: Roundabout at 120 km/h.

In addition, the NLT value of the vehicle was calculated (see Figure 23 and Figure 24). As can be seen in Table 7, the NLT value follows the same trend as in the two previous simulations. The difference between the controller designed in this research and the controller that did not consider the delay is 0.01 for the front axle and 0.02 for the rear axle. Regarding the uncontrolled system, the vehicle rolls over.

**Figure 25:**
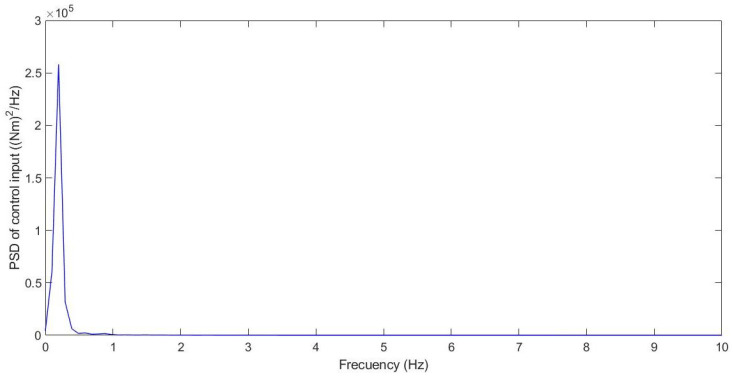
Frequency response for test 3: Roundabout at 120 km/h.

Additionally, the chattering phenomenon was analysed. This was caused by high-frequency changes in the control signal. This chatter may damage the actuator and compromise the control performance. Figure 25 shows the power spectral density (PSD) for the control input.

## 4. Conclusions

The novelty of this paper is the development of an LMI-based H${}_{\infty }$ output-feedback controller that compensates for the input and output delays in a Roll Stability Control (RSC) system. The Lyapunov stability theory and LMI were used to assure the stabilization for the considered system. Results show that the roll angle decreases when the controller proposed in this work is used.

To quantify the performance of the controller, the NLT was employed. Comparing the results obtained between the controller proposed in this work and a system with a standard H${}_{\infty }$ controller, the percentage of improvement of the NLT value for both axles were: 17.31% for lane change tests, 21.81% for the roundabout test, and 18.22% for a more severe roundabout test. Comparing the results obtained between the controller proposed in this work and a system without a controller, the percentage of improvement of the NLT value for both axles were: 34.48% for lane change tests, 30.55% for the roundabout test, and 59.01% for a more severe roundabout test. It was also seen that the NLT value only reaches the value of 1 in the roundabout, at a high speed, and without the controller system. In the cases with the controller proposed in this research, the NLT value reached 0.38.

Additionally, the chattering phenomenon was analysed. This was caused by high-frequency changes in the control signal. This chatter may damage the actuator and compromise the control performance. A frequency response was carried out for chatter study. Possible chattering problems were only detected for case 1 and case 2. In these cases, the frequency response of the control input was below 1.5 Hz, considered a low frequency, which did not cause damage to the actuators.

These results can be used in systems with problems that derive from the delay phenomenon. Furthermore, by using this controller with sensor fusion approaches, it may be possible to design real-time estimations and control for more secure driving. This research used the information provided by sensors that were already mounted on production vehicles such as IMUs.

## Figures and Tables

**Table 1:** Vehicle parameters.

Parameter	Description	Value
$I_{xx}$	Roll moment of inertia	500 kg m${}^{2}$
$m_{s}$	Sprung mass	1700 kg
$h_{cr}$	Sprung mass height about the roll axis	0.35 m
$C_{r}$	Total torsional damping	3538.08 N m/rad
$K_{r}$	Stiffness coefficient	18,438.02 N/m${}^{2}$
$l_{f}$	Distance from the Gravity Center to the front axle	1.51 m
$l_{r}$	Distance from the Gravity Center to the rear axle	1.99 m
*l*	Wheelbase	3.5 m
$t_{f}$	Half vehicle track, front axle	0.819 m
$t_{r}$	Half vehicle track, rear axle	0.819 m

**Table 2:** RMS and maximum errors of roll angle for test 1.

	RMS Error (°)	Maximum Error (°)
H${}_{\infty }$ Controller (Proposed in this research)	2.05	3.44
System Without Control	3.14	4.91
H${}_{\infty }$ Controller (Without delay in its design)	2.60	4.35

**Table 3:** Normalized Load Transfer for test 1.

	Normalized Load Transfer Front Axle	Normalized Load Transfer Rear Axle
H${}_{\infty }$ Controller (Proposed in this research)	0.25	0.24
System Without Control	0.36	0.34
H${}_{\infty }$ Controller (Without delay in its design)	0.32	0.31

**Table 4:** RMS and maximum errors of the roll angle for test 2.

	RMS Error (°)	Maximum Error (°)
H${}_{\infty }$ Controller (Proposed in this research)	1.56	2.35
System Without Control	2.36	3.48
H${}_{\infty }$ Controller (Without delay in its design)	1.87	2.81

**Table 5:** Normalized Load Transfer for test 2.

	Normalized Load Transfer Front Axle	Normalized Load Transfer Rear Axle
H${}_{\infty }$ Controller (Proposed in this research)	0.19	0.18
System Without Control	0.29	0.27
H${}_{\infty }$ Controller (Without delay in its design)	0.23	0.22

**Table 6:** RMS and maximum errors of roll angle for test 3.

	RMS Error (°)	Maximum Error (°)
H${}_{\infty }$ Controller (Proposed in this research)	1.75	4.53
System Without Control	4.72 (rollover)	9.81 (rollover)
H${}_{\infty }$ Controller (Without delay in its design)	2.14	5.52

**Table 7:** Normalized Load Transfer for test 3.

	Normalized Load Transfer Front Axle	Normalized Load Transfer Rear Axle
H${}_{\infty }$ Controller (Proposed in this research)	0.38	0.37
System Without Control	1	0.94
H${}_{\infty }$ Controller (Without delay in its design)	0.47	0.46

## Data Availability

Not applicable.

## Abbreviations

The following abbreviations are used in this manuscript:

LMI	Linear Matrix Inequality
RMS	Root Mean Square
RSC	Roll Stability Control
NCS	Networked Control Systems

## References

[1] Zhu B., Piao Q., Zhao J., Guo L. (2016). Integrated chassis control for vehicle rollover prevention with neural network time-to-rollover warning metrics. Adv. Mech. Eng..

[2] Yim S. (2012). Design of a robust controller for rollover prevention with active suspension and differential braking. J. Mech. Sci. Technol..

[3] Riofrio A., Boada M.J.L., Boada B.L., García-Pozuelo D. Fuzzy-Based Anti-Rollover Controller for a Heavy Duty Vehicle, using Active Suspension. Proceedings of the FIS ITA 2016 World Automotive Congress, BEXCO.

[4] Riofrio A., Sanz S., Boada M.J.L., Boada B.L. (2017). A LQR-Based Controller with Estimation of Road Bank for Improving Vehicle Lateral and Rollover Stability via Active Suspension. Sensors.

[5] Yoon J., Cho W., Yi K., Koo B. (2008). Unified Chassis Control for Vehicle Rollover Prevention. IFAC Proc..

[6] Yoon J., Cho W., Koo B., Yi K. (2009). Unified Chassis Control for Rollover Prevention and Lateral Stability. IEEE Trans. Veh. Technol..

[7] Rodríguez Licea M.A., Cervantes I. (2014). Robust Switched Predictive Braking Control for Rollover Prevention in Wheeled Vehicles. Math. Probl. Eng..

[8] Jaiwat P., Ohtsuka T. Stabilization of vehicle rollover by nonlinear model predictive control. Proceedings of the The SICE Annual Conference 2013.

[9] Chu D., Lu X.Y., Wu C., Hu Z., Zhong M. (2015). Smooth Sliding Mode Control for Vehicle Rollover Prevention Using Active Antiroll Suspension. Math. Probl. Eng..

[10] Hermans T., Ramaekers P., Denil J., Meulenaere P.D., Anthonis J. Incorporation of AUTOSAR in an Embedded Systems Development Process: A Case Study. Proceedings of the 2011 37th EUROMICRO Conference on Software Engineering and Advanced Applications.

[11] Sangiovanni-Vincentelli A., Di Natale M. (2007). Embedded System Design for Automotive Applications. Computer.

[12] Chakraborty S., Lukasiewycz M., Buckl C., Fahmy S., Chang N., Park S., Kim Y., Leteinturier P., Adlkofer H. Embedded systems and software challenges in electric vehicles. Proceedings of the Design, Automation Test in Europe Conference Exhibition.

[13] Guo J., Luo Y., Li K., Dai Y. (2018). Coordinated path-following and direct yaw-moment control of autonomous electric vehicles with sideslip angle estimation. Mech. Syst. Signal Proc..

[14] Strano S., Terzo M. (2017). Vehicle sideslip angle estimation via a Riccati equation based nonlinear filter. Meccanica.

[15] Zhang C., Chen Q., Qiu J. (2017). Robust H_∞_ filtering for vehicle sideslip angle estimation with sampled-data measurements. Trans. Inst. Meas. Control.

[16] Boada B.L., Boada M.J.L., Vargas-Melendez L., Diaz V. (2018). A robust observer based on H_∞_ filtering with parameter uncertainties combined with Neural Networks for estimation of vehicle roll angle. Mech. Syst. Signal Proc..

[17] Pajares Redondo J., Prieto González L., García Guzman J., Boada B.L., Díaz V. (2018). VEHIOT: Design and Evaluation of an IoT Architecture Based on Low-Cost Devices to Be Embedded in Production Vehicles. Sensors.

[18] Pajares Redondo J., Prieto González L., Montalvo Martinez M.M., García Guzman J., Sanz S., Boada M.J.L., Boada B.L. VEHIOT: Evaluation of Smartphones as Data Acquisition Systems to Reduce Risk Situations in Commercial Vehicles. Proceedings of the 2018 IEEE International Conference on Vehicular Electronics and Safety (ICVES).

[19] García Guzman J., Prieto González L., Pajares Redondo J., Sanz S., Boada B.L. (2018). Design of Low-Cost Vehicle Roll Angle Estimator Based on Kalman Filters and an IoT Architecture. Sensors.

[20] García Guzman J., Prieto González L., Pajares Redondo J., Montalvo Martinez M.M., Boada M.J.L. (2018). Real-Time Vehicle Roll Angle Estimation Based on Neural Networks in IoT Low-Cost Devices. Sensors.

[21] Farivar F., Sayad Haghighi M., Jolfaei A., Wen S. (2021). On the Security of Networked Control Systems in Smart Vehicle and Its Adaptive Cruise Control. IEEE Trans. Intell. Transp. Syst..

[22] Lyu W., Cheng X. (2017). A Novel Adaptive H_∞_ Filtering Method with Delay Compensation for the Transfer Alignment of Strapdown Inertial Navigation System. Sensors.

[23] Boada M.J.L., Boada B.L., Zhang H. (2021). Event-triggering H_∞_-based observer combined with NN for simultaneous estimation of vehicle sideslip and roll angles with network-induced delays. IEEE Trans. Control Syst. Technol..

[24] Shariati A., Taghirad H.D., Labibi B. (2012). Delay-Dependent H_∞_ Control of Linear Systems with Uncertain Input Delay Using State-Derivative Feedback. Sensors.

[25] Jin X., Yin G., Li Y., Li J. (2016). Stabilizing Vehicle Lateral Dynamics with Considerations of State Delay of AFS for Electric Vehicles via Robust Gain-Scheduling Control. Asian J. Control.

[26] Browne F., Rees B., Chiu G.T.C., Jain N. (2020). Iterative Learning Control With Time-Delay Compensation: An Application to Twin-Roll Strip Casting. IEEE Trans. Control Syst. Technol..

[27] Vargas-Melendez L., Boada B.L., Boada M.J.L., Gauchia A., Diaz V. (2017). Sensor Fusion Based on an Integrated Neural Network and Probability Density Function (PDF) Dual Kalman Filter for On-Line Estimation of Vehicle Parameters and States. Sensors.

[28] Chokor A., Talj R., Charara A., Doumiati M., Rabhi A. Rollover Prevention Using Active Suspension System. Proceedings of the 2017 IEEE 20th International Conference on Intelligent Transportation Systems (ITSC).

[29] Vargas-Melendez L., Boada B.L., Boada M.J.L., Gauchia A., Diaz V. (2016). A Sensor Fusion Method Based on an Integrated Neural Network and Kalman Filter for Vehicle Roll Angle Estimation. Sensors.

[30] Liu Y., Yang K., He X., Ji X. Active Steering and Anti-Roll Shared Control for Enhancing Roll Stability in Path Following of Autonomous Heavy Vehicle. Proceedings of the WCX SAE World Congress Experience.

